# MKP-1 attenuates LPS-induced blood-testis barrier dysfunction and inflammatory response through p38 and IκBα pathways

**DOI:** 10.18632/oncotarget.12823

**Published:** 2016-10-22

**Authors:** Yiqing Pan, Yue Liu, Li Wang, Feng Xue, Yanqin Hu, Ran Hu, Chen Xu

**Affiliations:** ^1^ Department of Anatomy, Histology and Embryology, Shanghai Jiao Tong University School of Medicine, Shanghai 200025, China; Shanghai Key Laboratory of Reproductive Medicine, Shanghai 200025, China; ^2^ Laboratory of Dermatology, Ruijin Hospital, Shanghai Jiao Tong University School of Medicine, Shanghai 200025, China; ^3^ Shanghai Institute of Immunology, Shanghai Jiao Tong University School of Medicine, Shanghai 200025, China

**Keywords:** MAP phosphatase-1, occludin, signaling transduction, inflammation, testicular immune privilege

## Abstract

Sertoli cells create a local tolerogenic microenvironment to maintain testicular immune privilege especially through the formation of a blood-testis barrier (BTB). However, the molecular mechanisms underlying the immune modulation function and BTB dynamics of Sertoli cells remained elusive. MAP phosphatase (MKP)-1 acts as a crucial negative regulator of the inflammatory response. Nevertheless, the role of MKP-1 in regulating Sertoli cells has not been elucidated. In this study, we have for the first time uncovered distinct cellular localization of MKP-1 in the cells at different stages of mouse testis, and the level of MKP-1 expression was significantly up-regulated by LPS-induced acute testis inflammation. In addition, MKP-1 staining was strongly detected in nuclei and peri-nuclear regions of cytoplasm in the Sertoli cells, and it was presented at Sertoli cell tight junctions (TJs) at stages VII-VIII after LPS treatment. Moreover, we demonstrated that MKP-1 was capable of attenuating LPS-induced decrease of occludin by interaction with p38 MAP kinase and IκBα molecules. Taken together, our data highlight that MKP-1 was an important endogenous suppressor of innate immune responses involved in the regulation of BTB barrier dynamic. This study thus might offer novel targets for treating inflammatory diseases in the testis.

## INTRODUCTION

Testis is an immune-privileged organ that protects auto-antigens from detrimental immune response and counteraction of invading microbial pathogens [1]. Meanwhile, testis can be infected by various microbial pathogens and exhibits special defense mechanisms [2]. An increasing evidence supports the fact that the breakdown of local testicular immune homeostasis may lead to temporary or permanent male infertility [3]. Recent studies suggest that the pattern recognition receptor (PRR) - mediated innate immune response in testicular cell is involved in testicular immune homeostasis and associated with inflammation-induced disruption of testicular function [4]. Further understanding of the mechanisms underlying testicular immune homeostasis could provide novel clues that would be useful for exploring male immunological subfertility.

Mitogen-activated protein kinase (MAPK) phosphatases (MKPs) belong to a family of dual specificity protein phosphatases with the function of dephosphorylating both phosphothreonine and phosphotyrosine residues of their substrates [5–7]. MKP-1, the first defined member of MKPs [8], has been regarded as an important regulator of innate immune response and tumorigenesis by deactivating both MAP kinases and NFκB pathway in immune cells [9–15]. Previous study has shown that MKP-1 transcripts are detected in E14.5 mouse embryonic reproductive system by RNA *in situ* hybridization [16]. Furthermore, it has been demonstrated that MKPs act as a negative regulator to modulate steroidogenesis in MA-10 Leydig cells. In these studies, the modulations of ERK1/2 MAP kinase signaling by MKPs on the hormonal action in Leydig cells were explored [17, 18]. However, it remains unknown about the role of MKP-1 in the seminiferous tubules during pathogen infection. Additionally, the mechanisms in which signaling pathway molecules participate remain to be defined.

Sertoli cells, the only somatic cell type within the seminiferous tubules, play a critical role in controlling testicular immune privilege status and local defense responses [19]. It has been demonstrated that Sertoli cells not only serve as a physical wall (by creating the BTB) but also possess the capacity to modulate the immune response [20, 21]. Furthermore, there are several excellent studies showing that MAP kinase pathway plays an important role in numerous male reproductive processes, including BTB dynamics, the germ cell-cycle progression and differentiation, and germ cell apoptosis in the seminiferous epithelium [22, 23]. On the other hand, some studies suggest that MAP kinase pathway is often involved in male reproductive dysfunction in some infection status [24]. Since activation of MAPK is associated with transduction downstream signals in the control of male reproductive processes, the inactivation of the MAPK is the same importance as its activation [25]. Consequently, it is of great significance to probe the role of MKP-1 in seminiferous epithelium, particularly, the immunological and physiological function of MKP-1 on BTB dynamic changes in infection status.

Occludin is the first identified integral membrane protein in the tight junction (TJ) [26], which plays an important role for proper TJ function in spermatogenesis [27–30]. Recent studies have shown that focal adhesion kinase (FAK) is structurally associated with occludin and regulates the structural interaction between occludin and ZO-1 in primary Sertoli cells [31]. In line with this regulatory mechanism, occludin has been indicated to serve as a substrate for a wide range of kinases and phosphatases in various pathophysiological contexts, illustrating that occludin may act as a “signaling” regulatory TJ protein [32]. In addition, it is interesting to note that BTB dysfunction is associated with activation of the MAP kinase pathway [22–24]. Although there is a general appreciation with the role of signaling pathway in the integrity of the BTB and the homeostasis of the seminiferous epithelium, it remains unclear how Sertoli cells contribute to creating a local tolerogenic environment by BTB and modulation of the immune response during pathogen infection. Therefore, this study was designed to elucidate the immune modulation function of MKP-1 in BTB dynamic especially on infection status.

In the present study, the expression pattern of MKP-1 was studied after LPS-induced acute testis inflammation. We revealed that induction of MKP-1 was correlated with the inactivation of MAP kinases and IκBα molecules in the LPS-stimulated Sertoli cells, suggesting that MKP-1 is a key endogenous suppressor of innate immune responses on testis infection status. We also identified that MKP-1 was capable of attenuating LPS-induced down-regulation of occludin by interaction with p38 MAP kinase and IκBα molecules. These results presented a novel mechanism of MKP-1 in modulating BTB dynamic. In addition, our findings may represent a novel mechanism for understanding the precise physiological relationship between immune regulation and TJ-integral membrane protein, e.g., occludin.

## RESULTS

### Distinct expression and localization of MKP-1 in the cells of mouse testes

#### Expression of MKP-1 in different stages of mouse testes

**Figure 1:**
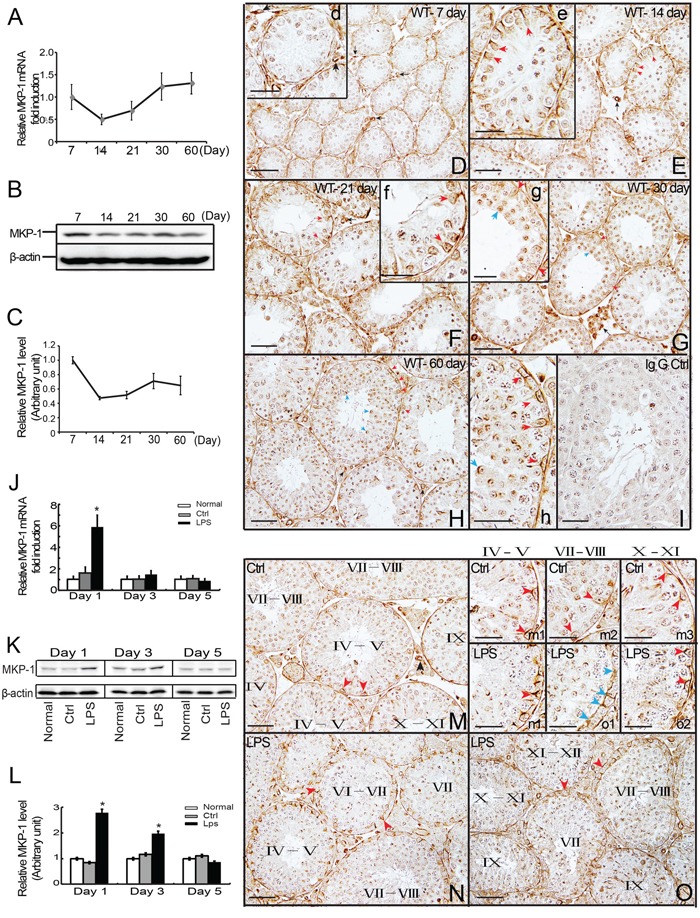
Expression and distribution of MKP-1 in the developing testes and LPS-induced acute testis inflammation Detection of MKP-1 expression in the developing testes (day 7 to day 60) was performed by realtime PCR **A**. and western blot **B-C**. analyses. Paraffin section of the testes of day 7 **D**., day 14 **E**., day 21 **F**., day 30 **G**. and day 60 **H**. were immunostained with anti-MKP-1 antibody. MKP-1 protein was detected in the Sertoli cells (red arrows), germ cells (blue arrows), some interstitial cells and blood vessel walls (black arrows). The Expression of *Mkp-1* in mRNA **J**. and protein **K, L**. levels during LPS-induced acute testis inflammation. Histological analyses showed MKP-1 expression by LPS stimuli **N** and **O**. compared with control **M**. In addition, positive staining at nuclei and perinuclear region of cytoplasm of Sertoli cells were detected at Sertoli cell (red arrows) and TJs (blue arrows) after LPS treatments (m1-m3 *vs*. n1, o1-o2). I: IgG control. Scale bar: 50 μm (D to I, M to O) and 20 μm (d to h, m1 to m3, n1, o1 and o2). * *p*<0.05.

To investigate the biological role of MKP-1 in male reproduction, we first compared the expression patterns of MKP-1 in different stages of mouse testes. We found that *Mkp-1* mRNA was expressed at a high level at postnatal day 7, day 21, day 30 and day 60, but decreased at day 14 (Figure 1A). We also examined MKP-1 protein levels in postnatal testes. Western blots showed MKP-1 expression at a high level at young and adult mouse testes, and its expression was decreased at puberty (Figure 1B, 1C). Compared with MKP-1 protein expression, *Mkp-1* mRNA level was much higher from day 30 to adult (day 60), indicating that MKP-1 translation may be modified at post-translational level in this stage.

#### Cellular localization of MKP-1 during testis development

To determine the spatial localization of MKP-1, immunohistochemistry (IHC) was performed using an antibody against MKP-1. MKP-1 was detected in the seminiferous tubules, some interstitial cells, and blood vessel walls in the adult mouse testes. Within the seminiferous tubules, MKP-1 was found at all stages of the epithelial cycles. The strongest staining was detected in the nuclei and perinuclear region of cytoplasm in the Sertoli cells and spermatids at day 60 of mouse testes. Various male germ cells, including spermatogonia, spermatocytes, were weakly stained (Figure 1H, 1h). The distributions of MKP-1 in mouse testes at day 14, day 21 and day 30 were similar to the adult pattern (Figure 1E-1G, e-g). Figure 1D, d showed MKP-1 staining in day 7 of mouse testes. In contrast to testis sections from day 14 to adult, MKP-1 was preferentially localized in peritubular cells, a subset of blood vessels, some interstitial cells, and the tunica. Meanwhile, MKP-1 protein was detected weakly in the seminiferous tubules, including Sertoli cells and germ cells.

### Changes of MKP-1 expression and localization during LPS-induced acute testis inflammation

#### Increased expression of MKP-1 in the testes after LPS treatments *in vivo*

Based on the previous studies of MKP-1 involved in the immune response, we asked whether there was the induction of MKP-1 during LPS-induced acute testis inflammation. Administration of LPS (5 mg/kg) resulted in a significant increase of MKP-1 expression in the testes at day 1 and day 3 after LPS treatments compared with control group (Figure 1K, 1L). In parallel, the transcription level of *Mkp-1* was greatly raised at day 1 to day 3 following LPS treatments (Figure 1J). The transcripts of *TNFα* and *IL-6* were enhanced in testes isolated from LPS-treated mice (data not shown), further confirming the local inflammatory response in the testes.

#### Stage-specific localization of MKP-1 at the Sertoli cells after LPS treatments *in vivo*

We next identified the cell types within the seminiferous tubules that expressed MKP-1 during the LPS-induced acute testis inflammation. Histological examination of MKP-1 expression in tubule cross sections after LPS treatments revealed a consistent correlation between the stage in seminiferous epithelial cycle and the level of MKP-1 expression. As shown in Figure 1M to O, the overall staining of the MKP-1 was increased by LPS stimulation. Furthermore, the spatial localization observation demonstrated that MKP-1 staining was strongly detected at nuclei and perinuclear region of cytoplasm in Sertoli cells, presented at Sertoli cell TJs at stages VII- VIII after LPS treatments (Figure 1-n1, o1, o2). Compared with the control, the staining of MKP-1 was much stronger in Sertoli cells at stages VII - VIII in LPS treated mice, indicating that MKP-1 may be involved in the BTB dysfunction during LPS-induced acute testis inflammation.

### Administration of LPS to mouse testes *in vivo* perturbed the BTB integrity

#### Increased the permeability of the BTB by the fluorescence tracer

**Figure 2:**
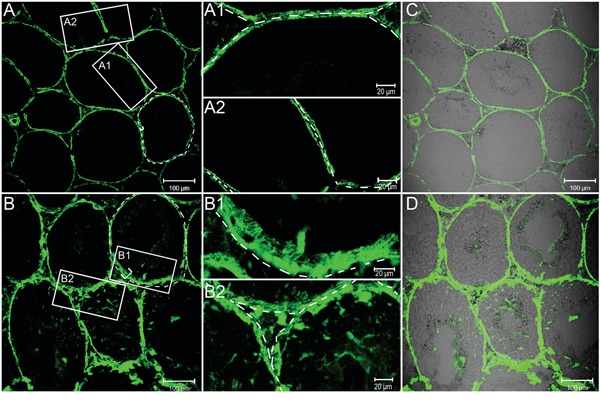
FITC fluorescence tracer assays to evaluate BTB permeability by LPS stimuli FITC fluorescence (green) was observed only in the basal area of seminiferous tubules of the control mice **A**., whereas in the mice during LPS-induced acute testis inflammation (5 day, **B**.) the tracer was also found in the adluminal compartment of seminiferous tubules. A1-A2, B1-B2: A magnified view of the boxed area in A and B, respectively. **C** and **D**. Same bright field images in A and B respectively were observed. Bars=100 μm (A - D) and 20 μm (A1, A2, B1 and B2).

It is well established that acute testicular inflammation, reproduced *in vivo* by the administration of bacterial LPS, has been shown to induce an array of pathophysiological response and characteristic symptom [2]. Previous studies also have shown that cytokines, such as TGF-β and TNFα, can reversibly disrupt the BTB *in vivo* [33–35]. To determine whether LPS administration could affect the permeability of the BTB, we next assessed the functional test of BTB integrity by monitoring the diffusion of FITC (Mr 389) from the systemic circulation to the seminiferous epithelium as described in Materials and Methods. We found that, in the testes from control mice, the FITC was limited to the interstitial space and unable to pass through the BTB located to near the basal membrane of the tunica propria (Figure 2A, see the white bracket). However, FITC was shown to diffuse into the adluminal compartment by day 5 following LPS treatment (Figure 2B).

#### Ultrastructural changes of the BTB by LPS-induced damage

**Figure 3:**
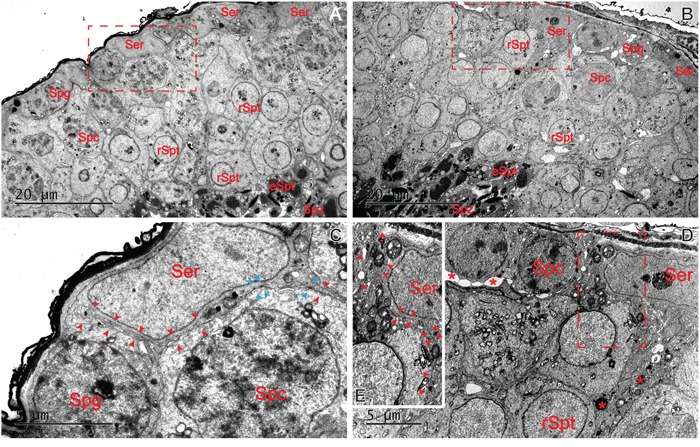
Ultrastructural changes at the BTB by LPS stimuli in adult mouse testes Adult mice were treated with a single dose of LPS (5 mg/kg) on day 0 and terminated on day 5 **B**. *versus* mice treated with control group **A**. The typical ultrastructural features of the BTB were found between two adjacent Sertoli cells lying on the basement membrane in the control testes (**C**. red arrowhead: actin filament bundles; blue arrowhead: TJ ultrastructures). After LPS treatments, changes in the spermatogenic epithelium were observed as a loosened organization. No obvious actin filament bundles or TJ ultrastructures was found (see red arrowheads in the boxed area in E). Sertoli cells appeared electron-dense and vacuolated, with an accumulation of large electron-dense bodies (**D**., red asterisks). Bars=20 μm (A and B) and 5 μm (C and D).

The LPS-induced damage to the BTB was further examined by electron microscopy. As shown in Figure 3A, 3C, the typical ultrastructural features of the BTB created by two adjacent Sertoli cells lying on the basement membrane (red arrowhead) were observed in the control testes. After LPS treatments, obvious ultrastructural damage was visible at the Sertoli-Sertoli cell interface (Figure 3B, 3D). The actin filament bundles and the associated endoplasmic reticulum (ER) at the basal ES that co-exists with TJ at the BTB became severely disorganized and defragment (see red arrowheads in the boxed area in Figure 3E) by day 5 after LPS treatment at a dose of 5 mg/kg when compared with control testes shown in Figure 3A, 3C (boxed area showing the typical ultrastructure of the BTB in normal adult mouse testes). In addition, changes observed in the spermatogenic epithelium were displayed as a loosened organization due to the disruption of the junctional complexes. Administration of LPS may also had a direct impact on Sertoli cells of transport vesicles. Sertoli cells appeared electron-dense and vacuolated, with an accumulation of large electron-dense bodies, probably representing phagolysosomes. Similar ultrastructural changes have been reported in Sertoli cells after the administration of proinflammatory cytokines [33].

### Changes of MKP-1 expression in Sertoli cells by LPS incubation

#### Increased expression of MKP-1 in Sertoli cells after LPS treatments

**Figure 4:**
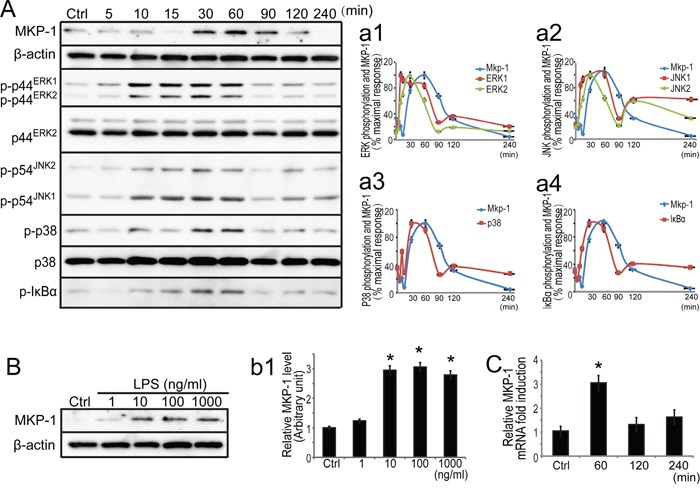
Expression of MKP-1 and related signaling molecules in primary Sertoli cells by LPS stimuli **A**. Primary Sertoli cells were either not stimulated or stimulated with LPS (100 ng/ml). Cells were harvested for protein after 0, 5, 10, 15, 30, 60, 90, 120 and 240 minutes, and cell lysates were subjected to western blot analysis for MKP-1, p-ERK1/2, ERK2, p-JNK1/2, p-p38, p38 and β-actin. **B**. Primary Sertoli cells were either not stimulated or treated with LPS (1, 10, 100 and 1000 ng/ml) for 60 minutes. Cells were harvested and cell lysates were subjected to western blot analysis for MKP-1 and β-actin. Immunoblots were scanned, and the intensity of bands was quantified by the image program. Data were normalized to the control and expressed as the percentage of maximum activation of stimulation (a1-a4) or the ratio was expressed as fold change with respect to the control sample (b1). **C**. Primary Sertoli cells were either not stimulated or treated with LPS (100 ng/ml) for 60, 120 and 240 minutes. Cells were harvested for RNA, and realtime PCR was carried out using primers specific to *Mkp-1* and *β-actin*. * *p*<0.05.

**Figure 5:**
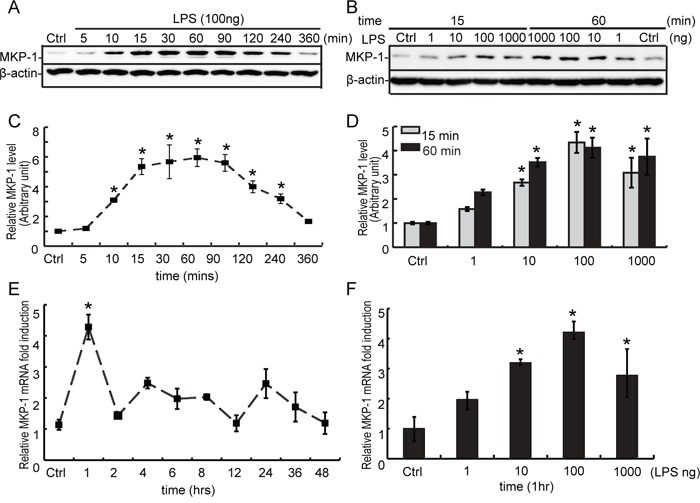
Expression of MKP-1 in TM4 Sertoli cells by LPS stimuli **A**. TM4 Sertoli cells were either not stimulated or stimulated with LPS (100 ng/ml). Cells were harvested for proteins after 0, 5, 10, 15, 30, 60, 90, 120, 240 and 360 minutes, and cell lysates were subjected to western blot analysis for MKP-1 and β-actin. **B**. TM4 Sertoli cells were either not stimulated or treated with LPS (1, 10, 100 and 1000 ng/ml) for 15 and 60 minutes. Cells were harvested and cell lysates were subjected to western blot analysis for MKP-1 and β-actin. Immunoblots were scanned, the intensity of bands was quantified by the image program. Data were normalized to the control, and the ratio was expressed as fold change in relation to the control sample **C** and **D**. **E**. TM4 Sertoli cells were either not stimulated or stimulated with LPS (100 ng/ml) for 1, 2, 4, 6, 8, 12, 24, 36 and 48 hours. Cells were harvested for RNA, and realtime PCR was carried out using primers specific to *Mkp-1* and *β-actin*. **F**. TM4 Sertoli cells were either not stimulated or treated with LPS (1, 10, 100 and 1000 ng/ml) for 60 minutes. Cells were harvested for RNA, and realtime PCR was performed using primers specific to *Mkp-1* and *β-actin*. * *p*<0.05.

Since Sertoli cells provide physical, nutritional, and hormonal support to the developing germ cells, our data described above revealed that MKP-1 was presented in mouse Sertoli cells and respond to acute testis inflammation, we thus assessed the role of MKP-1 in Sertoli cells by LPS incubation. We first examined the expression of MKP-1 in primary Sertoli cells by LPS incubation. In untreated primary Sertoli cells, the MKP-1 protein level was very low. In response to LPS stimulus (100 ng/ml), the induction of MKP-1 became evident at 30 minutes, greatly increased at 60 to 90 minutes, and returned to the basal levels at 240 minutes in primary Sertoli cells (Figure 4A). Increased concentrations of LPS (1~1000 ng/ml) caused a dose-dependent enhancement in primary Sertoli cells after a 60 minutes treatment period (Figure 4B). Realtime PCR also showed that LPS increased *Mkp-1* expression at 1 to 4 hours, and the peak was at 1 hour, as shown in Figure 4C. We also illustrated the LPS-induced MKP-1 activation in Sertoli cell line, namely TM4 Sertoli cells. Similar to the primary Sertoli cells, the expression of MKP-1 (both transcription and translation levels) was significantly induced *via* the dose- and time-dependent after LPS stimulus in the TM4 Sertoli cells (Figure 5).

#### Induction of MKP-1 was correlated with the inactivation of MAP kinases and IκBα in LPS-stimulated primary Sertoli cells

It has been reported that LPS can trigger a cascade of signaling events to activate the transcription factor NFκB and the MAP kinase, leading to production of a variety of proinflammatory cytokines, including TNFα and IL-6 in some immune cells [9–12]. Our realtime PCR analyses revealed that LPS induced *TNFα* and *IL-6* transcription in primary Sertoli cells (data not shown). The kinetics of MAP kinase activation stimulated with LPS were examined by western blots using antibodies specifically recognizing the phosphorylated ERK1/2, JNK1/2 and p38 in primary Sertoli cells (Figure 4A). The activated forms of ERK1/2, JNK1/2 and p38 revealed maximal activation by LPS at 30 minutes, and their activation was decreased and returned to nearly basal level by 90 minutes (Figure 4A, a1-a3). Since there is convincing evidence that NFκB pathway may present a possible mechanism of action occurring at the induction of MKP-1 by inflammation stimuli [13], we further explored the relationship between MKP-1 molecule and the NFκB pathway in primary Sertoli cell by LPS stimuli. It is well known that IκBα inactivated NFκB by trapping NFκB complex in the cytoplasm under resting conditions and activated by phosphorylation (typical at serine 32 and 36) upon cell stimulation [36]. Western blot analysis for the Ser 32/36-phosphorylated form of IκBα revealed maximal activation by LPS at 30~60 minutes, and decreased to the basal levels by 90 minutes (Figure 4A and a4). Considered together, inflammatory stimuli by LPS initiated signaling molecules including both MAP kinases and IκBα molecules in primary Sertoli cells.

Meanwhile, in response to LPS stimulation, MKP-1 protein was obviously induced at 60~90 minutes in primary Sertoli cells (Figure 4A). The reciprocal relationship between MKP-1 and the signaling molecules supported the notion that MKP-1 may play an important role in the feedback control of MAP kinases and IκBα molecules in Sertoli cells by inflammation stimuli.

#### LPS-induced MKP-1 expression was involved in both MAP kinases and NFκB pathways in Sertoli cells

**Figure 6:**
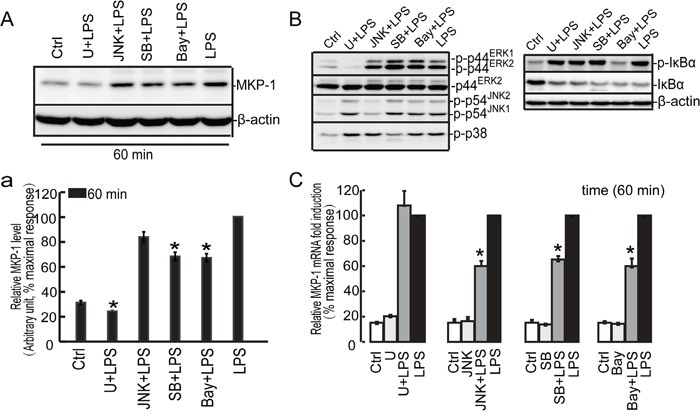
Effect of the U0126, SB203580, JNKII and Bay 11-7082 on LPS induced MKP-1 expression in the TM4 Sertoli cells TM4 Sertoli cells were incubated with U0126 (10 μM), SB203580 (10 μM), JNKII(10μM) and Bay 11-7082 (10 μM) as indicated for 30 minutes prior to stimulation with LPS (100 ng/ml) for 60 minutes. Cells were harvested for RNA and protein, realtime PCR **C**. and western blot **A**, **B**. analysis were carried out using primers and antibodies specific to MKP-1 and β-actin. Data were normalized to the control and expressed as the percentage of maximum activation of stimulation **a**. and **C**. * *p*<0.05.

To understand the signaling cascade of MKP-1 induction in LPS-activated Sertoli cells, we utilized U0126 (MEK-ERK1/2 inhibitor), JNKII (JNK inhibitor), SB203580 (p38 inhibitor) and Bay 11-7082 (IκBα inhibitor) to assess MKP-1 production. Since the peak of LPS-induced MKP-1 expression occurred at 60 minutes at mRNA and protein levels, this period was used to study the effect of these pharmacological inhibitors on the induction of MKP-1 (Figure 6 and Supplementary data). Treatment of Sertoli cell line, TM4 cells with SB203580 and Bay 11-7082 (10 μM, pretreated for 30 minutes) were capable of inhibiting LPS-induced MKP-1 production (both mRNA and protein levels) at 60 minutes (about 0.6~0.7 fold). U0126 (10 μM, pretreated for 30 minutes) almost blocked LPS-induced MKP-1 expression at protein level, whereas it had little effect on *Mkp-1* transcription. Compared with other compounds, JNKII (10 μM, pretreated for 30 minutes) also had some effect on *Mkp-1* expression at mRNA level, resulting in ~40% reduction in *Mkp-1* transcript. The effects of these pharmacological inhibitors on MKP-1 expression revealed that ERK1/2, p38 and IκBα molecules were critically involved in MKP-1 induction in response to LPS stimulation in TM4 Sertoli cells.

**Figure 7:**
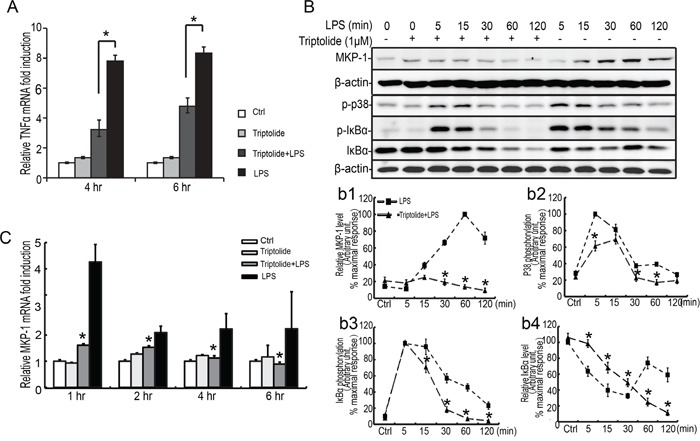
Effect of triptolide on LPS induced TNFα expression and p38 and IκBα phosphorylation in the TM4 Sertoli cells TM4 Sertoli cells were incubated with triptolide for 30 minutes prior to stimulation with LPS (100 ng/ml) for 1, 2, 4 and 6 hours. Cells were harvested for RNA, and realtime PCR was carried out using primers specific to *TNFα, Mkp-1* and *β-actin* **A** and **C**. **B**. TM4 Sertoli cells were incubated with triptolide as indicated for 30 minutes prior to stimulation with LPS (100 ng/ml) for 5, 15, 30, 60 and 120 minutes. Cell lysates were subjected to western blot analysis for p-p38, p38, p-IκBα, IκBα, and β-actin. Data were normalized to the control and expressed as the percentage of maximum activation of stimulation (b1-b4). * *p*<0.05.

Triptolide is a major active component derived from the medicinal plant *Tripterygium wilfordii* Hook F (TWHF). The anti-inflammatory effect of triptolide in some cell models has been demonstrated in some groups [37, 38]. To further explore the mechanism of MKP-1 induction in LPS-activated Sertoli cells, we utilized triptolide to assess MKP-1 and related signaling molecules expression. Triptolide (1 μM, pretreated for 30 minutes) could inhibit LPS induced *TNFα* expression in TM4 Sertoli cells (Figure 7A). Meanwhile, administration of triptolide inhibited LPS-induced phosphorylation of p38 and IκBα molecules and almost blocked MKP-1 expression (Figure 7B, 7C). These data indicated that both p38 and IκBα were important upstream molecules for the induction of MKP-1 in Sertoli cells by LPS stimulating.

### LPS-induced MKP-1 was involved in regulation of occludin

#### LPS induced down-regulation of occludin expression and accelerated occludin internalization

**Figure 8:**
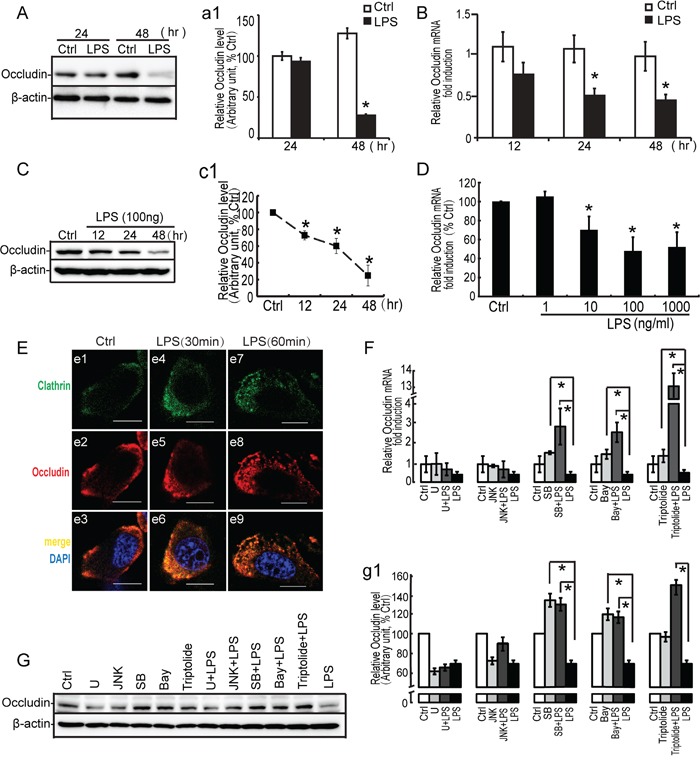
Effect of LPS on occludin expression, localization and study of signal molecules related to occludin expression by LPS Primary Sertoli cells **A**, **B**. and TM4 Sertoli cells **C**, **D**. were either not stimulated or stimulated with LPS (100 ng/ml). Cells were harvested for RNA or protein after 0, 12, 24 and 48 hours. (A, C) Cell lysates were subjected to western blot analysis for occludin and β-actin. Immunoblots were scanned, and the intensity of bands was quantified by the image program. Data were normalized to the control and expressed as the percentage of maximum activation of stimulation (a1, c1). (B, D) Cells were harvested for RNA, and realtime PCR was carried out using primers specific to *occludin* and *β-actin*. **E**. LPS (100 ng/ml) was added to the medium for 30 and 60 minutes. Cells were immunostained with an anti-occludin antibody (red fluorescence), and internalized protein vesicles were found to colocalize with clathrin (green fluorescence) at 60 minutes after LPS treatment. TM4 Sertoli cells were incubated with U0126 (10 μM), SB203580 (10 μM), JNK II(10μM) and Bay 11-7082 (10 μM) as indicated for 30 minutes prior to stimulation with LPS (100 ng/ml). Cells were harvested for RNA and protein, realtime PCR **F**. and western blot **G**. analysis were carried out using primers and antibodies specific to occludin and β-actin. Data were normalized to the control and the ratio was expressed as fold change (F) or the percentage of maximum activation of stimulation (g1) with respect to the control sample. * *p*<0.05.

Occludin can seal the intercellular space of Sertoli cells and maintain the function of BTB, but the function of occludin remains elusive. Recent studies have shown that occludin is required for cytokine-induced regulation of tight junction barrier [28, 35, 39, 40]. To investigate whether occludin plays a role in LPS-induced barrier disruption, we evaluated the expression of occludin in Sertoli cells by LPS stimuli. As shown in Figure 8A, 8B, the higher expression of occludin was detected in the control group, and a significant decrease in occludin expression (both in mRNA and protein levels) was observed in primary Sertoli cells at 24 to 48 hours by LPS incubation. A similar change pattern for occludin was detected in Sertoli cell line, TM4 cells, compared to the primary Sertoli cells (Figure 8C, 8D). Moreover, the effect of LPS on occludin localization was also observed. Occludin (red fluorescence) was localized primarily to the cell-cell interface with weak cytoplasmic and nuclei staining in vehicle-treated Sertoli cells (Figure 8E, e2). After LPS treatment, clathrin (green fluorescence)-mediated endocytosis was increased (Figure 8E, e4, e7), which led to the decline of occludin in Sertoli cell membrane (Figure 8E, e5, e8). Furthermore, internalized protein clathrin was found to be co-localized with occludin at 30~60 minutes after LPS treatment, as shown in Figure 8E, e6, e9. These data indicated that LPS was capable of down-regulating occludin expression and modifying its distribution *via* enhanced endocytosis.

#### LPS-induced down-regulation of occludin was modulated by p38 and IκBα molecules

Recent evidence demonstrated that some protein kinases could modulate the expression and localization of occludin [39, 41–43]. To determine whether occludin decrease was associated with LPS-induced activation of signaling molecules, we exploited the pharmacological inhibitors for MAP kinases and IκBα molecules to assess occludin expression. Since mRNA and protein analyses for occludin revealed that a significant decrease was observed at 24 to 48 hours after LPS treatment, this period was employed to assess the effect of these inhibitors on occludin expression (Figure 8F, 8G). LPS-induced down-regulation of occludin in TM4 Sertoli cells was completely blocked by SB203580 (p38 inhibitor) and Bay11-7082 (IκBα inhibitor) pretreatment (10 μM for 30 minutes). In addition, in response to inhibitor alone (SB203580 or Bay 11-7082), we observed a mild increase of occludin expression by western blots and PCR analyses. In fact, the effect of U0126 on occludin expression was also predominant. Administration of ERK inhibition alone or LPS suppressed the expression of occludin significantly both at mRNA and protein levels. However, prior to incubation with U0126 could not restore the reduction of occludin by LPS in Sertoli cells. In addition, JNK inhibitor revealed modest effect on regulating occludin expression. Therefore, these data demonstrated that down-regulation of occludin by LPS stimuli in Sertoli cells was more dependent on p38 MAPK and IκBα molecules.

To further explore the mechanism of MKP-1 induction on regulating occludin, TM4 Sertoli cells were treated with anti-inflammatory drugs, triptolide to block the phosphorylation of p38 and IκBα by LPS stimuli (Figure 7). In the presence of triptolide (1 μM, pretreated for 30 minutes), the repression of occludin was significantly reversed in TM4 Sertoli cells by LPS incubation. Meanwhile, administration of triptolide alone had little effect on regulating occludin expression (Figure 8F, 8G). These data showed that the effect of triptolide (1 μM) on occludin occurred only by LPS stimulating, which suggested that MKP-1 induction was involved in modulation of occludin expression in stress state.

#### Mkp-1 knockdown lead to impaired progression of occludin via p38 and IκBα molecules

**Figure 9:**
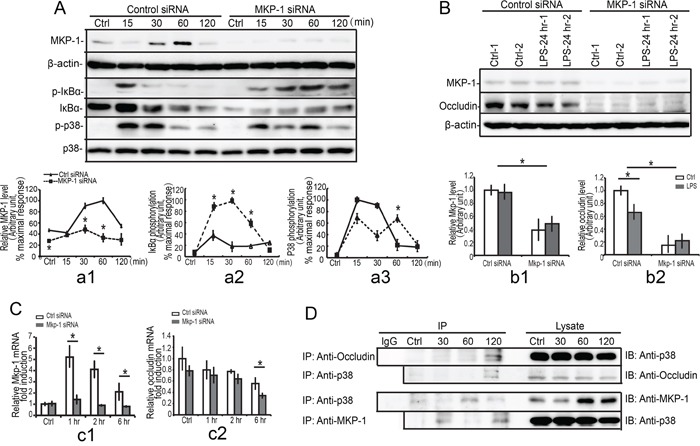
Interaction of occludin, p38 with MKP-1 TM4 *Mkp-1* silencing cells and TM4 control cells were either not stimulated or stimulated with LPS (100 ng/ml). Cells were harvested for protein after 0, 15, 30, 60 and 120 minutes (short timepoint, **A**.) or 24 hours (long timepoint, **B**.). Cell lysates were subjected to western blot analysis for MKP-1, phospho-IκBα (p-IκBα), total-IκBα (IκBα), phospho-p38 (p-p38), total-p38 (p38), and β-actin (short timepoint, A) or MKP-1, occludin, and β-actin (long timepoint, B). Immunoblots were scanned, and the intensity of bands was quantified by the image program. Data were normalized to the control and expressed as the percentage of maximum activation or fold change of stimulation (a1-a3, b1-b2). **C**. *Mkp-1* siRNA or control siRNA TM4 Sertoli cells were either not stimulated or stimulated with LPS (100 ng/ml). Cells were harvested for RNA after 1, 2 and 6 hours. Cells were harvested for RNA, and realtime PCR was carried out using primers specific to *Mkp-1* (c1), *occludin* (c2) and *β-actin*. Data were normalized to the control and the ratio was expressed as fold change relative to the control sample. **D**. TM4 Sertoli cells were either not stimulated or stimulated with LPS. Cells were harvested for protein after 30, 60 and 120 minutes, and MKP-1 was immunoprecipitated (IP) from cell lysates and analyzed by western blotting for presence of p38, occludin and MKP-1. *p<0.05.

To further investigate the role of LPS-induced MKP-1 in the repression of occludin, we assessed the expression of occludin in *Mkp-1* knockdown TM4 Sertoli cells. In the resting cells, the expression of MKP-1 was very low; LPS stimulation resulted in an increase in MKP-1 induction (30 to 60 minutes), whereas no MKP-1 was detected in *Mkp-1* siRNA cells when stimulating by LPS both at mRNA and protein levels (Figure 9C, c1 and a1). Meanwhile, the higher expression of occludin was detected in the resting control siRNA cells, and a significant decrease in occludin expression was observed by LPS incubation. In addition, knockdown of *Mkp-1* by RNAi was found to induce a drastic down-regulation of occludin both mRNA and protein levels at 6 hours and 24 hours respectively compared with the control siRNA group (Figure 9C, c2 and b2). These results indicated that the loss of MKP-1 function resulted in impaired progression of occludin.

Our previous data demonstrated that down-regulation of occludin by LPS stimuli in Sertoli cells was more dependent on p38 MAPK and IκBα molecules, and thus we explored the molecular mechanisms of MKP-1 induction in regulating of occludin. As shown in Figure 9A, a3, *Mkp-1* siRNA cells exhibited a substantial increase (15 to 30 minutes) and a prolonged (60 to 120 minutes) p38 phosphorylation compared with control siRNA cells. Meanwhile, MKP-1 was also involved in attenuation of IκBα phosphorylation induced by LPS. Western blot analyses revealed that maximal expression of phospho-IκBα was detected after 15 minutes of LPS stimulation and it was gradually returned to the basal level till 30 minutes. *Mkp-1* knockdown resulted in a prolonged (30 to 120 minutes) IκBα activation (Figure 9A, a2). In addition, the total IκBα protein levels were also decreased when treated with LPS (Figure 9A). These data indicated that LPS-induced decrease of occludin was modulated by MKP-1 through p38 and IkBa molecules.

#### Interaction of occludin, p38 with MKP-1

Our present data suggested that LPS-induced decrease of occludin could be modulated by MKP-1 through p38. Thus we explored the possibility of a direct interaction between occludin and p38 in Sertoli cells by LPS. After stimulating the TM4 Sertoli cells by LPS at different times, immunoprecipitations were performed using antibody against occludin. The immunoprecipitates were fractioned by SDS-PAGE and immunoblotted with antibody against p38. In the resting cells, p38 was not linked to occludin. After stimulated with LPS, the p38 was efficiently precipitated with occludin at 120 minutes (Figure 9D). We also assessed the ability of p38 to bind occludin directly, and occludin was sufficiently detected in immunoprecipitates of p38 at 120 minutes. It is not the case in the resting cells, indicating that there was a specific affinity between occludin and p38 after stimulated with LPS.

We next examined the interaction between MKP-1 and p38 in Sertoli cells. As shown in Figure 9D, immune-precipitation revealed that p38 was efficiently precipitated with MKP-1 at 30 to 120 minutes after LPS stimulation. Although various signaling cascades that were involved in the inflammatory responses undergo changes in the expression patterns after LPS infection, the relationship among MKP-1, p38 and occludin displayed distinct interaction each other in the spatio-temporal manner. Same peak time was exhibited at 120 minutes when linking to MKP-1 in Sertoli cells by LPS. Taken together, our data indicated that MKP-1 could control p38 by direct interaction and thus it might modulate occludin *via* p38-MKP-1 complex.

## DISCUSSION

MKP-1 is an important regulator of innate immune response by deactivating both MAP kinases and NFκB pathway [9–13]. The role of MKP-1 in the seminiferous tubules has not yet been elucidated. The immunological and physiological function of MKP-1 on BTB dynamic changes during pathogen infection are poorly understood. The present study demonstrated the first evidence showing the expression pattern of MKP-1 during LPS-induced acute testis inflammation. Furthermore, we uncovered the relationship between MKP-1 and occludin by LPS stimuli, and our study provided a novel mechanism between local immunosuppressive molecules and Sertoli cell junction dynamics during LPS-induced acute testis inflammation.

Testicular immune privilege is attributed to the coordination of systemic immune tolerance, local physical structure, and active local immunosuppression. Not only are numerous immunological contents in the interstitial area of the testis (e.g., macrophages, dendritic cells), but there is considerable evidence supporting that Sertoli cells play a critical role in modulating the immune response in the testis [19–21]. As examples, one crucial function of Sertoli cells is the formation of the BTB. Increasing evidence suggests that the BTB is a dynamically regulated structure [22-24, 35]. Previous studies have shown that BTB dysfunction is closely associated with the activation of the MAP kinases and NFκB pathways in some infection status [24, 35, 40]. In our study, administration of LPS to mouse testes could perturb the BTB integrity and activation of MAP kinases and IκBα molecules. Meanwhile, negative regulator of the inflammatory response, MKP-1 was remarkably induced in the testes in response to LPS stimulation. Histological analyses showed that MKP-1 was widely expressed in developing and adult testis tissues and became stronger by LPS stimuli. In addition, intense staining at nuclei and perinuclear region of cytoplasm of the Sertoli cells were detected at Sertoli cell TJs after LPS treatment. Furthermore, the stage-specific localization (stages VII-VIII) of MKP-1 in the Sertoli cells was observed in response to LPS stimuli *in vivo*. These data implicated that MKP-1 could be a feedback control of both MAP kinases and NFκB pathway in Sertoli cells and may participate in BTB dynamic changes during LPS-induced acute testis inflammation.

Occludin is the first identified integral trans-membrane protein within TJ and it assumes to play an important role in the regulation of TJ formation, structure, and function. Unlike other TJ proteins, occludin has a relative long cytoplasmic tail and it is regulated tightly by phosphorylation [41]. It has been shown that occludin is mediated largely by the membrane protein associated signaling molecules at the junction *via* protein phosphorylation and dephosphorylation [35, 39, 40]. In the present study, gene silencing of *Mkp-1* by RNAi in TM4 Sertoli cells led to a significant increase in LPS-induced down-regulation of occludin. These data provided evidence that MKP-1, the protein phosphatase, could modulate the expression of occludin by LPS stimuli in Sertoli cells. Thus, we subsequently investigate the mechanisms of LPS-induced MKP-1 in the repression of occludin. Our findings suggested that MKP-1 was potently induced and attenuated LPS-induced decrease of occludin through mechanisms mediated, in a large part, by the p38 pathway. This notion was supported by the observations as follows. First, MKP-1 was involved with p38 pathway in Sertoli cells in response to LPS stimulation. Second, knockdown of *Mkp-1* in TM4 Sertoli cells resulted in a substantially increase p38 phosphorylation. Additionally, down-regulation of occludin by LPS stimuli in Sertoli cells was more dependent on p38 molecule. Furthermore, immunoprecipitation assays revealed a direct interaction between occludin and p38 in TM4 Sertoli cells by LPS. Meanwhile, MKP-1 can modulate p38 by direct binding. These finding are consistent with the conclusion by Huber and Cheng, C. Y. that an interaction of p38 with occludin after treatment of LPS [22, 23, 44]. Considering that induction of MKP-1 also correlated with inactivation of ERK and JNK kinases in Sertoli cells, the effects of these pharmacology inhibitors on occludin expression were also assessed in Sertoli cells. In fact, occludin expression was decreased partly by MEK/ERK inhibitor alone, which indicated that the importance of ERK pathway in the regulation of occludin, preferentially under resting conditions. On the other hand, administration of JNK inhibitor did not show a significant inhibitory effect on occludin expression. These observations suggested that MKP-1 could act as an important negative regulator that attenuated LPS-induced down-regulation of occludin through p38 MAPK pathway.

Another interesting topic in our study was the interaction among MKP-1, IκBα with occludin by LPS stimuli. In the current study, a block in IκBα phosphorylation by the pharmacological inhibitor prevented the LPS-induced decreased in occludin expression in Sertoli cells. These data agreed with previous studies indicating occludin function could be regulated in a manner dependent on NF-κB [45, 46]. Meanwhile, it has been suggested that IκBα is associated with MKP-1 dependent negative regulation, which is attributed to modulate the immune response [13, 14]. In a kinetic analysis of IκBα phosphorylation, we found primarily an effect of MKP-1 deficiency on the up-regulation of IκBα activation that led to markedly increased phospho-IκBα levels in Sertoli cells by LPS stimulation. We also explored the interaction between MKP-1 and IκBα at the same way. Unfortunately, very weak band was detected by in immune-precipitates, suggesting that the interaction between MKP-1 and IκBα may be more complicated.

Taken together, we proposed that various signaling components were involved in the inflammatory responses in the testes by LPS stimulation and they cooperated each other and assumed a distinct spatio-temporal regulation. MKP-1 acted as an important negative regulator for the signaling cascades, strictly controlled the duration and intensity of the inflammatory response to LPS, and maintained the local testicular immune homeostasis.

**Figure 10:**
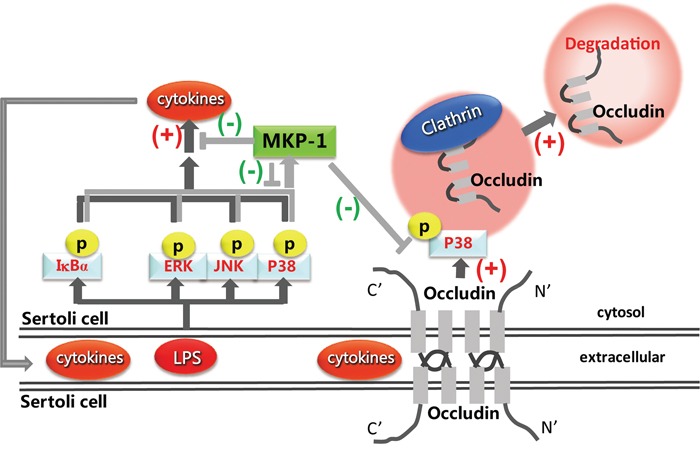
Proposed model illustrated that MKP-1 attenuated LPS-induced Blood-Testis Barrier dysfunction and inflammatory response through p38 and IκBα pathways

In summary, we have for the first time demonstrated the regulation effect of MKP-1 on TJ protein, occludin by LPS stimulation in mouse Sertoli cells. MKP-1 is likely an important regulator in attenuating LPS-induced down-regulation of occludin by interaction with p38 and IκBα molecules (Figure 10). These findings illustrated that inactivation of the signal transduction cascades was as important as its activation in regulation of occludin function during pathogen infection in Sertoli cells, which could restrain the potentially devastating action and prevented self-destruction, and maintain the local testicular immune homeostasis.

## MATERIALS AND METHODS

### Reagents and antibodies

Lipopolysaccharide (LPS, *Escherichia coli O55:B5*) was purchased from Sigma-Aldrich (St Louis, MO). U0126, SB203580, JNK Inhibitor II and Bay117082 (Calbiochem Corp, San Diego, CA) were dissolved in DMSO, and the final concentration of DMSO was less than 0.1%. Triptolide- *Tripterygium wilfordii* was purchased from Calbiochem Corp (La Jolla, CA). Occludin antibody was obtained from Invitrogen (Camarillo, CA). Clathrin was purchased from BD Transduction Laboratories. The antibodies against phospho-ERK1/2 (Thr202/Tyr204), phospho-JNK (Thr183/Tyr185), phospho-p38 (Thr180/Tyr182), and total p38 were purchased from Cell Signaling Technology. Total-ERK2 and MKP-1 antibodies were obtained from Santa Cruz Biotechnology. β-actin was purchased from Sigma. Secondary HRP-conjugated antibodies were from Santa Cruz Biotechnology. Dulbecco's modified Eagle's medium, penicillin and streptomycin were obtained from Invitrogen Life Technologies. All other reagents were purchased from Sigma, unless indicated otherwise.

### Animals and *in vivo* LPS treatments

Male C57BL6 mice were hosted in the Association for Assessment and Accreditation of Laboratory Animal Care-certified SPF facility of the Shanghai Jiao Tong University, School of Medicine. Animals were kept under standardized conditions of lighting (12 hours Light: 12 hours Dark) with free access to water and standard food pellets. Animals were routinely manipulated daily during the 1 week before the experiment to reduce stress levels. The experimental protocols were approved by the Shanghai Jiao Tong University, School of Medicine Animal Care Protocol.

Male adult C57BL6 mice were used for *in vivo* LPS challenge experiments. Mice (8~10 weeks, ~24 g b.w.) were randomly assigned to the control group or treatment groups. Initially, mice were received a single intraperitoneal (IP) injection of sterile saline alone (~200 μl, control) or with saline containing LPS (~200 μl) at a dose of 5 mg/kg body weight, since this was a widely used dose of LPS for pathogen challenge [2], each group containing 6 animals. The animals were sacrificed by cervical dislocation, and testes were removed after injection (1, 3 and 5 days), immediately until further analysis.

### Immunohistochemistry

Mice at 7, 14, 21, 30 and 60 days *pp* were sacrificed by cervical dislocation, and testes were immediately removed and fixed with Bouin's solutions at room temperature overnight (day 0 was birth date). We used 4 mice per group. Then, 5 μm-thick serial paraffin sections were prepared. A number of paraffin sections were stained with haematoxylin staining using standard protocols, and other sections were immunostained using the following procedure. For antigen retrieval, the sections were incubated in buffered citrate (pH 6.0) for 15 minutes at 105°C for MKP-1. The samples were then soaked in methanol containing 0.3% H_2_O_2_ to neutralize endogenous peroxidase activity. Sections blocked in 10% normal rabbit serum for 30 minutes at room temperature were incubated with rabbit anti-MKP-1 (1:200; Santa Cruz Biotechnology) at 4°C overnight. On the following day, the sections were incubated with a polyclonal goat anti-rabbit IgG antibody. The sections were visualized using a Histostain-Plus Kit (LAB-SA Detection; System Invitrogen Life Technologies) according to the manufacturer's instructions for the secondary antibody and subsequent steps. Testis seminiferous epithelium stage analysis was performed as previously described [47].

### *In vivo* BTB integrity assay

The permeability of the BTB was performed pursuant to the protocol as earlier described [33, 48] to assess the ability of an intact BTB to block the diffusion of a fluorescence tag (inulin-FITC, Mr 4.6 kDa) across the barrier from the basal to the adluminal compartment in the seminiferous epithelium. Briefly, male mice were anesthetized, and then 200 μl of 1 mg/ml fluorescein isothiocyanate isomer I (Sigma-Aldrich, USA), freshly diluted in saline, were injected into the caudal vein of mice. About 90 minutes thereafter, the mice were euthanized and their testes were immediately removed and embedded in OCT (Sakura). Cryostat sections with 8μm thickness were obtained in a cryostat microtome and fluorescence images were acquired by a laser scanning confocal microscope (LSCM, Carl Zeiss LSM-510, Jena, Germany).

### Electron microscopy analysis

For electron microscopy, the small pieces of testicular tissue were fixed with glutaraldehyde, postfixed in reduced osmium, dehydrated, and embedded as previously described [48]. The sections were analyzed with a transmission electron microscope (PHILIPS CM-120, Eindhoven, Netherlands) at 80 kV.

### Cell culture and treatment

#### Primary Sertoli cell isolation and culture

Testicular Sertoli cells were isolated from ~10- to 14-day-old mice and cultured as described previously [33]. Briefly, testes were sequentially treated with 1mg/ml collagenase in DMEM containing 25 U/ml DNase I twice at room temperature for 10 minutes, and then with 2 mg/ml hyaluronidase and 1mg/ml collagenase in DMEM containing 25 U/ml DNase I at 37°C for 20 minutes with agitation. After filtration using a 35-μm filter, cells were cultured in serum-free Dulbecco's modified Eagle's medium (DMEM) for 24 hours at 32°C to allow attachment, followed by a change of medium to deplete unattached germ cells. The purity of the resulting Sertoli cells is routinely > 90%, as assessed by FCM and immunofluorescence using antibody directed against testis specific androgen binding protein (also called sex hormone-binding globulin, SHBG, from Santa Cruz Biotechnology) for Sertoli cells.

#### Sertoli cell line

The Sertoli cell line, namely TM4 cell, was obtained from American Tissue Culture Collection (ATCC, Rockville, MD) and cultured in DMEM supplemented with 10% FBS, 100U/ml of penicillin G and 100 μg/ml streptomycin in a humidified 37°C incubator.

#### *In vitro* LPS incubation

Prior to experimentation, cells were seeded on six well plate (2×10^5^ per well), incubated overnight, and changed to fresh medium with penicillin/streptomycin. LPS was dissolved in serum-free medium and added to the medium at the indicated concentrations. Triptolide was dissolved in DMSO and added to the culture media at the concentrations indicated. In the inhibition experiments, the MEK/ERK inhibitor-U0126, the p38 inhibitor-SB203580, the JNK inhibitor-JNK Inhibitor II and the IκBα inhibitor-BAY 11-7082 were added to the medium at a final concentration of 10 μM at 30 minutes before the addition of LPS.

### siRNA (small interfering RNA) of *Mkp-1*

TM4 Sertoli cells were seeded on 24-well plate (0.5×10^5^ per well), incubated overnight, and changed to fresh medium with no penicillin/streptomycin. The siRNA vectors with or without nucleotide sequences targeting *Mkp-1* were obtained from Santa Cruz Biotechnology (Lafayette, CO, USA) as previously described [49]. A final concentration of 60 pmol *Mkp-1* siRNA or control siRNA was transfected into the TM4 Sertoli cells utilizing X-tremeGENE HP DNA Transfection Reagent (Roche Molecular Biochemicals, Mannheim, Germany) following the manufacturer's protocol. To determine the efficiency of gene silencing, the whole cell lysate of the above transfected cells was used for western blotting as described below and probed using specific antibody for MKP-1. At 72 hours after transfection, cells were treated with LPS or inhibitors for the indicated time before being lysed for used for each experiment.

### RNA isolation, cDNA synthesis, and real time PCR

Total RNA from the indicated mice and cell lysates was isolated using TRIzol® reagent (Invitrogen) according to the manufacturer's recommendations, and reverse transcription reactions were prepared using 1 μg of RNA to obtain cDNA (Qiagen). The resultant cDNA was diluted 1:3 in RNAse-free water. Realtime quantitative PCR (Q-PCR) was performed using ABI 7700 Sequence Detection System (Applied Biosystems) as described [48]. The sequence of primers used were as follows: mouse *Mkp-1*: sense, 5'-ACC ATC TGC CTT GCT TAC CTT-3'; antisence: 5'- AGC ACC TGG GAC TCA AAC TG-3'; mouse *TNFα*: sense, 5'- GAC CCT CAC ACT CAG ATC ATC TTC-3'; antisence: 5'- CAC GTA GTC GGG GCA GCC TTG-3'; mouse *occludin*: sense, 5'- CCT ACT CCT CCA ATG GCA AA-3'; antisence: 5'- CTC TTG CCC TTT CCT GCT TT-3'.mouse *β-actin*: sense, 5'- GAA GAG CTA TGA GCT GCC TGA-3'; antisence: 5'-CAG CAC TGT GTT GGC ATA GAG -3'.

### Protein preparation, immunoprecipitation, and western blotting

Mice from the indicated mice (n = 3 for each time point) at different ages were sacrificed, and testes were removed immediately, frozen in the liquid nitrogen, and stored at -80°C until use. Testis and cell lysates were prepared using lysis buffer containing 10 mM HEPES (pH 7.4), 50 mM β-glycerophosphote, 1% Triton X-100, 1% NP-40, 10% glycerol, 2 mM EDTA, 2 mM EGTA, 1 mM DTT, 10 mM NaF, 1 mM Na_3_VO_4_ and complete protease inhibitor cocktail (Roche Diagnostics). The lysates were centrifuged and the supernatants were boiled in SDS loading buffer. Protein concentrations were determined by the Bradford method, using the Coomassie Plus Assay Reagent (Pierce). For immunoprecipitation, cell lysates were incubated with appropriate amount of antibody overnight and then precipitated following absorption onto protein A/G-agarose. Precipitates were washed three times, separated by SDS-PAGE, and transferred onto PVDF membranes (Millipore) which was then incubated in TBST buffer (150 mM NaCl, 20 mM Tris-HCl, and 0.02% Tween 20, pH 7.6) containing 5% non-fat milk. Western blots were performed utilizing 30~50 μg of lysate protein, 8~10% SDS-PAGE gels depending on the apparent relative molecular mass of the target protein to be investigated. Western blot analysis was conducted by using ECL reagent (Pierce). The membranes were probed with the following antibodies with appropriate dilutions: MKP-1, occludin, phospho-ERK1/2, phospho-JNK and phospho-p38. Phospho-ERK1/2, phospho-JNK and phospho-p38 were stripped and re-probed with antibodies against total-ERK2 or total-p38, respectively, to confirm equal protein loading.

### Immunofluorescence

Immunofluorescence was assessed was performed according to method as previously described [33, 48]. In brief, TM4 Sertoli cells were cultured on round cover glasses with a cell density of 0.1 × 10^5^ cells/cm^2^ and incubated in 24-well plate. LPS was added to the medium at the indicated concentrations. After treatment, cells were fixed with 4% paraformaldehyde (PFA) (wt/vol) in PBS (10 mM sodium phosphate, 0.15 M NaCl, pH 7.4 at 22°C), permeabilized with 0.1% Triton X-100, and then blocked with 10% normal goal serum (Invitrogen) for 30 minutes at room temperature followed by a subsequent overnight incubation at 4°C with rabbit anti-occludin antibodies (1:200 dilution) and mouse anti-clathrin antibodies (1:200 dilution). After washing, secondary antibodies conjugated with either Alexa fluor® 488 and Alexa fluor® 555 (Invitrogen) of 1:200 dilution were used. For negative control, normal rabbit or mouse IgG were used in place of primary antibodies. Finally, the fluorescence-stained sections were viewed under a laser scanning confocal microscope (Carl Zeiss LSM-510, Jena, Germany). Immunofluorescence experiments were repeated at least three times.

### Statistical analysis

Results are expressed as mean ± SEM. Statistical significance of differences between mean values was assessed with Student's *t*-test for unpaired data and analysis of variance analysis whenever required. All reported significance levels represent two-tailed p values. A p value of <0.05 was used to indicate statistical significance. Each experiment was performed in triplicate or quadruplicates (*n* = 3 or 4) and all experiments were repeated at least three times to ensure reproducibility.

## SUPPLEMENTARY MATERIALS FIGURES


