# Supplementary material for: MKP-1 attenuates LPS-induced blood-testis barrier dysfunction and inflammatory response through p38 and IκBα pathways
Source: Oncotarget. 2016 Oct 22;7(51):84907–23. doi: 10.18632/oncotarget.12823 (PMC5356708; doi:10.18632/oncotarget.12823)
Supplement: Supplementary file 1 [file oncotarget-07-84907-s001.pdf]

MKP-1 attenuates LPS-induced blood-testis barrier dysfunction and inflammatory response through p38 and IκBα pathways

SUPPLEMENTARY FIGURE

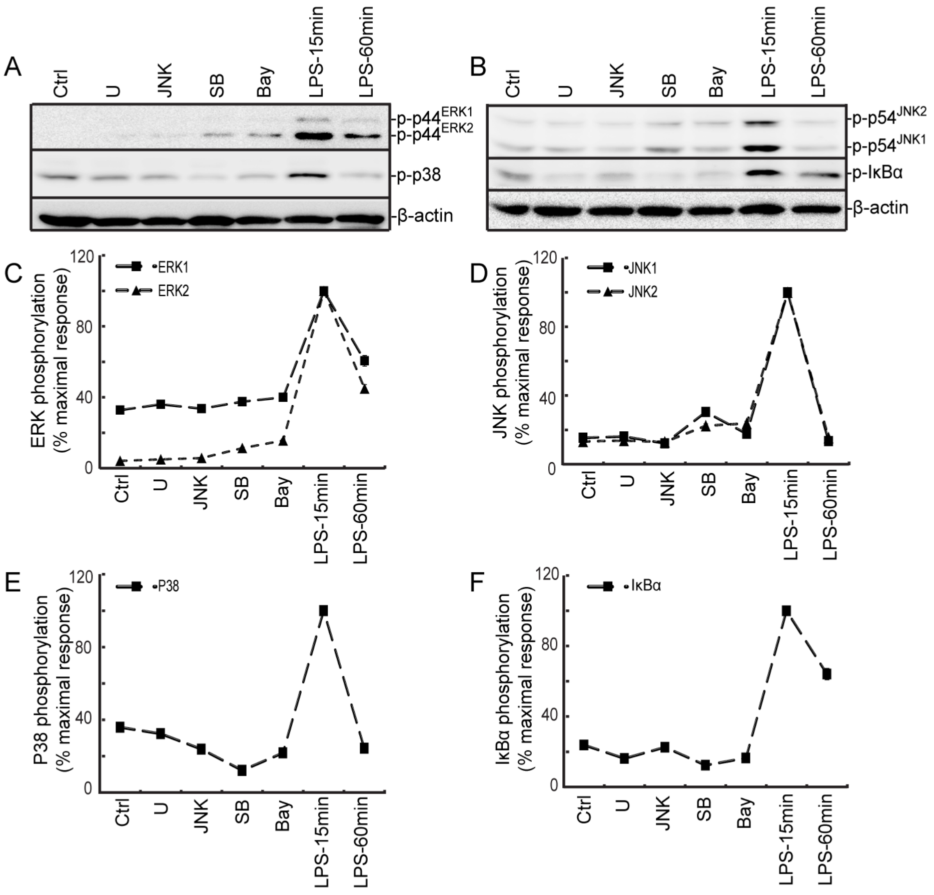

Supplementary Figure S1: Inhibitory effect of pharmacological inhibitors on the induction of MAP kinases and NFκB molecules. A. B. TM4 Sertoli cells were incubated with U0126 (10 μM), SB203580 (10 μM), JNKII(10μM) and Bay 11-7082 (10 μM) as indicated for 30 minutes prior to stimulation with LPS (100 ng/ml) for 15 and 60 minutes. Cell lysates were subjected to western blot analysis for p-ERK1/2, ERK2, p-JNK1/2, p-p38, p38, p-IκBα, IκBα and β-actin. Data were normalized to the control and expressed as the percentage of maximum activation of stimulation **C-F.** * $p<0.05$.
